# Anti-Nectin4 monoclonal antibodies attenuate airway inflammation and epithelial barrier dysfunction in asthma

**DOI:** 10.3389/fphar.2026.1931755

**Published:** 2026-10-07

**Authors:** Pureun-Haneul Lee, MinHyeok An, SangMin Yoon, An-Soo Jang

**Affiliations:** Department of Internal Medicine, Soonchunhyang University Bucheon Hospital, Bucheon, Republic of Korea

**Keywords:** airway inflammation, antibody, asthma, drug evaluation, Nectin4

## Abstract

**Objective:**

Nectin4 is an epithelial cell-adhesion molecule that may be involved in airway inflammatory responses. However, the effects of Nectin4 targeting in asthma has not yet been investigated.

**Methods:**

Here we engineered anti-Nectin4 monoclonal antibody, N-1T1C8Ab or N-1T3H9Ab, their effects on airway epithelial dysfunction and inflammation. Dermatophagoides pteronyssinus 1 (*Der p* 1)–stimulated normal human bronchial epithelial (NHBE) cells and ovalbumin-induced asthmatic BALB/c mice were used as experimental models.

**Results:**

Anti-Nectin4 antibody treatment restored epithelial barrier dysfunction and was associated with changes in IL-33-related, MyD88/IRAK, and Afadin/Rac1 signaling in NHBE cells. In the murine asthma model, administration of N-1T1C8Ab or N-1T3H9Ab reduced airway inflammation, airway resistance, and immune cell infiltration in bronchoalveolar lavage fluid. Histological analysis demonstrated decreased inflammatory cell infiltration, goblet cell hyperplasia, and fibrosis in lung tissues. Anti-Nectin4 antibody treatment was also associated with modulation of IL-33/MyD88-related signaling components.

**Conclusion:**

These findings indicate that anti-Nectin4 monoclonal antibodies attenuate airway inflammation and improve epithelial barrier function. Further preclinical studies are warranted to determine the potential of Nectin4 targeting in asthma.

## Introduction

Asthma is a chronic inflammatory respiratory disease that causes repeated bronchoconstriction and airflow limitation, affecting 262 million children and adults worldwide in 2019 with high mortality in low and lower-middle-income countries (Global Initiative For Chronic Obstructive Lung Disease, 2023). It is characterized by cardinal symptoms of airway hyperresponsiveness (AHR), cough, chest tightness, and breathing difficulties (Louis et al., 2022), manifesting with varying severity and impact on quality of life (Garg et al., 2024).

Asthma treatment primarily focuses on controlling symptoms and preventing flare-ups by reducing inflammation and managing airway constriction. The main treatments currently used for asthma are corticosteroids and controller medications, such as inhaled long-acting bronchodilators (LABA) (Global Initiative for Asthma, 2023). However, Long-term and high-dose use of corticosteroids can lead to various side effects, such as pneumonia, opportunistic infections, diabetes and lipid disorders (Bleecker et al., 2020; Luskin et al., 2016). Therefore, there is a need to develop safer and more potent alternative drugs that can effectively manage asthma symptoms over long periods, even at high doses. In recent years, there has been a shift from steroid treatment to an increased uptake of biologics with six Food and Drug Administration (FDA)-approved (Inselman et al., 2020). While available biologics effectively reduce asthma symptoms and exacerbation rates to varying extents (Menzies-Gow et al., 2021), often target specific phenotypes for higher response rates, and in some cases, requiring patient testing to confirm eligibility for certain subpopulations (Kim et al., 2017; Ross et al., 2024).

Nectin4 is a transmembrane cell adhesion molecule from the Nectins family that regulates cellular activities by promoting the formation of intercellular junctions (Takai et al., 2008a). Nectin4 has been identified as a marker for cancer relapse and metastasis and is associated with poor prognosis across various carcinomas (Takano et al., 2009; Fabre-Lafay et al., 2007; Deng et al., 2019; Nishiwada et al., 2015). Furthermore, our previous research demonstrated that Nectin4 is highly expressed in the lung tissue of asthmatic mice and that elevated levels of this protein are also present in the blood of asthma patients (Lee et al., 2022). We found that, compared to wild-type asthmatic mice, the knockdown of Nectin4 significantly alters airway responsiveness and inflammation and affects the expression of Afadin as well as the activated forms of Src and Rac1 (Lee et al., 2022). Nectin4 contributes to cell growth, angiogenesis, proliferation, and apoptosis through the involvement of the Rac1 and PI3K/Akt pathways (Siddharth et al., 2017; Zhang et al., 2016).

IL-33 is an epithelial-derived alarmin cytokine that plays an important role in initiating and amplifying airway inflammation. It is mainly released from epithelial barrier tissues such as the bronchus, nasopharynx, and skin in response to cellular stress or injury (Cayrol and Girard, 2018; Molofsky et al., 2015; Pichery et al., 2012). IL-33 binds to its receptor ST2 together with the co-receptor IL-1 receptor accessory protein (IL-1RAcP), forming a signaling complex that activates downstream MyD88-dependent pathways (Liu et al., 2013; Günther et al., 2017; Liew et al., 2016). Activation of this pathway promotes inflammatory responses by inducing cytokine production and immune cell activation, thereby contributing to the pathogenesis of allergic airway inflammation (Saluja et al., 2015; Saluja et al., 2014; Cho et al., 2012; Schneider et al., 2009; Schmitz et al., 2005).

Given that airway epithelial cells are major sources of IL-33 and that Nectin4 is involved in epithelial junctional signaling, we hypothesized that Nectin4 modulation may be associated with IL-33-related inflammatory responses, either directly or indirectly through changes in epithelial barrier function. Therefore, this study investigated the effects of Nectin4-targeting antibodies on epithelial barrier function, IL-33-related signaling, and airway inflammation.

To date, only one nectin4-specific drug-Enfortumab Vedotin-has been approved on urothelial carcinoma (Powles et al., 2021) and the clinical trials that test novel therapeutics are only five. Moreover, the potential role of Nectin4 as a therapeutic target in asthma has not been investigated. In this study, we evaluated the preclinical effects of anti-Nectin4 monoclonal antibodies on airway epithelial dysfunction and inflammation using bronchial epithelial cells *in vitro* and an experimental asthma model *in vivo*.

## Materials and methods

### Chemicals and reagents

The antibodies used were mouse anti-MyD88, IRAK1, IRAK4, TRAF6, Nectin4 antibodies (purchased from Novus), and antibodies against Erk1/2, p-Erk1/2, p38, p-p38, JNK, p-JNK, NF-κB, and p-NF-κB (from Cell Signaling Technology), and Afadin (from Invitrogen), Rac1 (from BD biosciences), Rac1-GTP (from Neweast biosciences). Mouse interleukin (IL)-4, 5, mouse tumor necrosis factor (TNF)-α, Interferon gamma (IFN-γ), IL-33, ST2, IL-1Racp enzyme-linked immunosorbent assay (ELISA) kits were purchased from R&D Systems and NeoBiolab. Recombinant human and mouse Nectin4 proteins were purchased from R&D Systems. All assays were conducted according to the manufacturers’ protocols, unless indicated otherwise.

### Development of phage display-derived Nectin4-specific antibodies, N-1T1C8Ab or N-1T3H9Ab

The scFv clones were isolated from a human phage library (Hu#1, YntoAb) by biopanning using purified recombinant human Nectin4 (rhNectin4; 2659-N4, R&D Systems). The recombinant human Nectin4 antigen was immobilized on immunotubes and coated in sodium bicarbonate buffer (pH 9.6) overnight at 4 °C. After coating, the tubes were blocked with 3% skim milk in PBST (PBS containing 0.05% Tween-20) for 1 h at room temperature. Phage particles (∼10^15^ CFU) were incubated with the immobilized antigen. After incubation, unbound phages were removed by washing five times with PBST, and bound phages were eluted using 100 mM triethylamine and immediately neutralized with 1 M Tris-HCl (pH 7.4). The eluted phages were used to infect *Escherichia coli* at 37 °C for 1 h, and infected bacteria were plated on LB agar plates containing ampicillin and incubated overnight at 37 °C. The following day, bacterial colonies were inoculated into SB medium containing ampicillin and cultured to an optical density (OD600) of approximately 0.6. Helper phage (VCSM13) was added for phage amplification. Phages were harvested from culture supernatants and purified by polyethylene glycol (PEG8000)/NaCl precipitation, resuspended in PBS, and used for subsequent rounds of biopanning. A total of three rounds of panning were performed to enrich antigen-specific phage clones.

Individual bacterial colonies were isolated, and monoclonal binders were screened by ELISA against recombinant human Nectin4. scFv proteins were expressed in the periplasm of *E. coli* following IPTG induction, and periplasmic extracts were used for ELISA screening. ELISA signals were measured using antigen-coated plates (rhNectin4) and non-coated wells (background), and the differential optical density (ΔOD) between the two conditions was used for clone selection. Clones meeting the predefined criteria (target OD > 1.0 and background OD < 0.2) were considered positive binders. Selected antigen-specific clones were further analyzed by Sanger sequencing. Sequence analysis revealed redundant sequences, and representative clones were selected based on sequence quality and diversity. Ultimately, two clones (N-1T1C8 and N-1T3H9) were selected for further characterization.

Nectin4-specific scFv binders were inserted into scFv-Fc vectors and transiently expressed in Expi293F cells (Thermo Fisher Scientific, United States). The scFv-Fc antibodies were secreted into the culture supernatant, and monoclonal scFv-Fc antibodies were purified by protein A affinity chromatography, followed by dialysis against PBS (pH 7.4). The purity of N-1T1C8Ab and N-1T3H9Ab was greater than 95% as determined by SDS-PAGE followed by Coomassie Brilliant Blue staining. The typical antibody yield ranged from 1.5 to 3.0 mg per 100 mL of culture. After dialysis with PBS, the antibody concentration was measured using a NanoDrop 2000 spectrophotometer (ThermoFisher Scientific, MA, United States).

### Determination of binding activity of antibodies

Human (2659-N4; R&D system) and mouse (3116-N4; R&D system) Nectin4 antigen were coated into ELISA plates (1 μg/mL, 50 μL/well) in sodium bicarbonate buffer (pH 9.6) at 2 °C–8 °C overnight using 96-well plates (Corning). After coating, plates were blocked with 3% skim milk in PBST (PBS containing 0.05% Tween-20) for 1 h at room temperature. N-1T1C8Ab or N-1T3H9Ab were diluted from 0 μg/mL to 2000 μg/mL and incubated at room temperature for 2 h. Plates were washed three to five times with PBST between each step. Goat anti-human IgG HRP was diluted to 1:20,000, added to the microplate, and incubated for 1 h. The TMB working solution was added to the microplate plate and incubated for 5 min at room temperature. Hydrochloric acid (1 M) was used to stop the reaction. A microplate reader (Thermo, VARIOSKAN LUX) was used to read the absorption value at 450 nm. The four-parameter curve was fitted using SoftMax software, and the sample’s half maximal effective concentration (EC50) value was calculated.

### Cell culture

Normal human bronchial primary epithelial cells (NHBE) were cultured in bronchial epithelial cell growth medium supplemented with the BEGM BulletKit™ (Bronchial Epithelial Cell Growth Medium) (Lonza) at 37 °C under a 5% CO_2_ atmosphere. Before treatment, cells were incubated and converged to 70%–80% in the cell culture flasks. Cells were treated as follows: *Der p* 1: Pretreated with 10 μg/mL for 8 h before adding N-1T1C8Ab or N-1T3H9Ab. N-1T1C8Ab or N-1T3H9Ab: 1, 10, 100 μg/mL for 48 h. Cell proliferation was determined using the MTT cell viability assay kit (Roche). NHBE cells were seeded onto 96-well plates, and after treatment, 10 μL MTT was added to each well and incubated for 4 h. 100 μL Solubilization solution was added to each well for overnight. Cell viability was determined by measuring the absorbance of each well at 550 nm using a microplate absorbance reader. Trans-epithelial electrical resistance (TEER) was measured using an Epithelial Volt/Ohm Meter (EVOM) (EVOM2, World Precision Instruments, FL). Baseline TEER was measured before any treatment and defined as 0 h. TEER was subsequently measured at 4, 8, 24, and 48 h after antibody administration. TEER was calculated by subtracting the resistance of a blank insert from the measured resistance and multiplying the resulting value by the surface area of the insert (1.12 cm^2^). TEER values were expressed as Ω·cm^2^.

### Animals

Specific-pathogen-free BALB/c (DBL, Eumseong-gun, Korea) mice were maintained under pathogen-free conditions at 20 °C ± 2 °C, 50%–70% humidity, and 12 h light/dark cycle. Mice were randomly assigned to four groups of 6–8 mice per group: control; ovalbumin (OVA); OVA + N-1T1C8Ab; OVA + N-1T3H9Ab groups. The mice were stimulated with intraperitoneal injection of 50 μg of OVA emulsified in 10 mg of aluminum hydroxide (Al(OH)_3_) in 0.1 mL of phosphate-buffered saline (PBS) on days 0 and 14, and were then exposed to 5% OVA aerosol every day from day 21–23. N-1T1C8Ab or N-1T3H9Ab were respectively administered by intratracheal injection on days 18–23, with PBS used as a vehicle control. Twenty-four hours after the last intranasal challenge, airway resistance was evaluated, bronchoalveolar lavage fluid (BALF) and blood were collected, and lung tissue was harvested for protein extraction and hematoxylin and eosin (H&E) and Masson’s trichrome staining. All animal procedures followed a protocol approved by the Institutional Animal Care and Use Committee of Soonchunhyang University Bucheon Hospital (SCHBC-animal-2020-11).

### Measuring mouse lung mechanics

Lung mechanics in the mice were determined using the flexiVent apparatus (SCIREQ, Montreal, QC, Canada). The mice were anesthetized with 2.5 mg/kg tiletamine and xylazine (Zoletil™; Bayer Korea, Seoul, Korea). After sedation, a tracheostomy was performed, and an 18-gauge cannula was inserted and connected to a computer-controlled ventilator. The mice were ventilated with room air with a tidal volume of 0.2 mL at a rate of 150 breaths/min. After 2 min of ventilation, the quasi-static compliance was measured as reported previously (Ishii et al., 2005).

### Western blotting

For Western blot analysis, the protein concentration was determined using a BCA protein assay kit (Thermo Fisher Scientific, MA, United States). Proteins were separated and transferred to polyvinylidene fluoride (PVDF) membranes, which were blocked with 5% bovine serum albumin (BSA) in 0.1% Tween 20 in Tris-buffered saline (TBS), and incubated with anti- MyD88, IRAK1, IRAK4, TRAF6, Erk1/2, p-Erk1/2, p38, p-p38, JNK, p-JNK, NF-κB, p-NF-κB, nectin4, afadin, rac1, and rac1-gtp antibodies (4 °C, overnight) followed by horseradish peroxidase (HRP)-conjugated secondary antibodies. Bands were visualized using the WEST-ZOL plus Western blotting Detection System (iNtRON, Seongnam, Korea). The relative abundance of proteins was determined by quantitative densitometry with normalization to β-actin (Sigma-Aldrich, MO, United States).

### Histological assessment of lung tissue

A portion of each left lung was fixed in 4% (v/v) buffered paraformaldehyde and embedded in paraffin. Lung sections were stained with H&E for histopathological analyses or with Masson’s trichrome to evaluate the collagen content and distribution, or with periodic acid-Schiff (PAS) to detect mucus secretion or with Toluidine stain to detect mast cell. Images were analyzed using ImageJ software (National Institutes of Health, Bethesda, MD, United States).

For double immunofluorescence staining, lung sections were incubated with primary antibodies against Nectin4 and Rac1-GTP overnight at 4 °C. The sections were then incubated with Alexa Fluor 488-conjugated goat anti-rabbit IgG (H + L) and PE-conjugated donkey anti-mouse IgG (H + L) (Abcam, United Kingdom) at a dilution of 1:200 for 2 h at room temperature. Nuclei were counterstained with DAPI, and fluorescence images were acquired using a confocal microscope (Leica, Germany).

### Enzyme-linked immunosorbent assay

IL-4, IL-5, TNF-α, IFN-γ, IL-33, ST2, IL-1Racp levels in lung lysates from mice were determined using ELISA. The lower detection limit was set at 2 pg/mL (IL-4), 7 pg/mL (IL-5), 1.88 pg/mL (TNF-α), 2 pg/mL (IFN-γ), 6.85 pg/mL (IL-33), 0.014 ng/mL (ST2), 0.1 ng/mL (IL-1Racp) based on the manufacturer’s recommendations.

### Statistical analyses

Statistical analyses were performed with SPSS 22.0 software. Data are expressed as means ± standard errors of the mean (SEMs). For the TEER time-course experiment, two-way repeated-measures ANOVA was used to evaluate the effects of treatment, time, and treatment × time interaction, followed by Bonferroni-adjusted pairwise comparisons. The differences between groups were determined using the nonparametric Kruskal–Wallis test, followed by Dunn’s *post hoc* test and Mann- Whitney U test. P < 0.05 was deemed to indicate statistical significance.

## Results

### Anti-Nectin4 monoclonal antibodies demonstrate binding affinity and restore barrier function without cytotoxicity in NHBE cells treated with *Der p* 1

We evaluated the binding affinities of N-1T1C8Ab and N-1T3H9Ab to recombinant human and mouse Nectin4 by ELISA. For human Nectin4, the EC50 values of N-1T1C8Ab and N-1T3H9Ab were 12.9556 and 37.1964 μg/mL, respectively (Figure 1A). For mouse Nectin4, the corresponding EC50 values were 20.7807 and 77.7941 μg/mL, respectively (Figure 1A). These results demonstrate that both antibodies bind to human and mouse Nectin4, confirming their cross-reactivity with mouse Nectin4. To assess cytotoxicity, NHBE cells treated with *Der p* 1 were exposed to varying concentrations of N-1T1C8Ab or N-1T3H9Ab (1–100 μg/mL) for 48 h (Figure 1B). No significant differences in cell viability were observed following treatment with either antibody compared with the controls. Next, we investigated the effects of N-1T1C8Ab and N-1T3H9Ab on epithelial barrier function by measuring TEER in *Der p* 1-treated NHBE cells. Repeated-measures analysis revealed significant treatment × time interactions for both N-1T1C8Ab and N-1T3H9Ab (both p < 0.001). At 48 h, treatment with either antibody at 1, 10, or 100 μg/mL significantly attenuated the *Der p* 1-induced reduction in TEER (Bonferroni-adjusted p < 0.05; Figure 1C). Together, these findings indicate that both antibodies bind Nectin4 without detectable cytotoxicity and are associated with improved epithelial barrier function following *Der p* 1 exposure.

**FIGURE 1:**
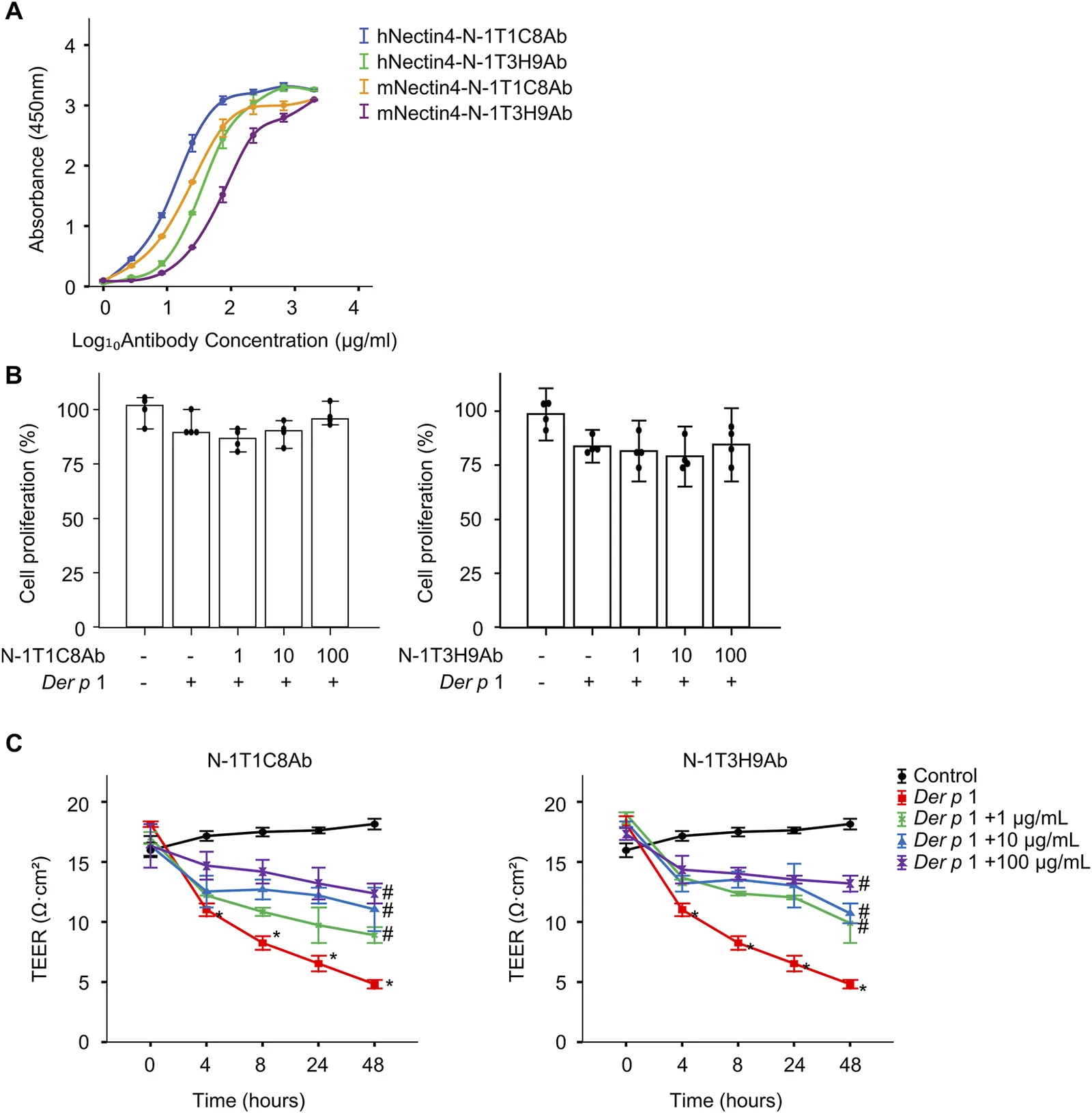
Anti-Nectin4 monoclonal antibody repairs the airway epithelial barrier in NHBE cells. **(A)** Binding activities of N-1T1C8Ab or N-1T3H9Ab to human and mouse Nectin4 as measured by ELISA. **(B)** Cell viability of NHBE cells after treatment with N-1T1C8Ab or N-1T3H9Ab for 48 h following *Der p* 1 application for 8 h. **(C)** Trans-epithelial electrical resistance (TEER) of NHBE cells after treatment with N-1T1C8Ab or N-1T3H9Ab following *Der p* 1 application for 8 h. Data are means ± SEMs from three independent experiments. *p < 0.05 compared to control, ^#^p < 0.05 compared to *Der p* 1.

### Anti-Nectin4 monoclonal antibodies modulate IL-33-related signaling components in NHBE cells treated with *Der p* 1

Following *Der p* 1 exposure, the expression levels of IL-33 and its receptor components, ST2 and IL-1 receptor accessory protein (IL-1RAcP), were significantly increased in NHBE cells (p < 0.05; Figures 2A,B). Treatment with N-1T1C8Ab or N-1T3H9Ab showed variable effects on these IL-33-related signaling components, with reductions observed for selected markers and antibody concentrations, although the changes in IL-33 did not reach statistical significance. Overall, the effects of anti-Nectin4 antibody treatment on IL-33-related signaling were marker- and concentration-dependent rather than uniformly suppressive.

**FIGURE 2:**
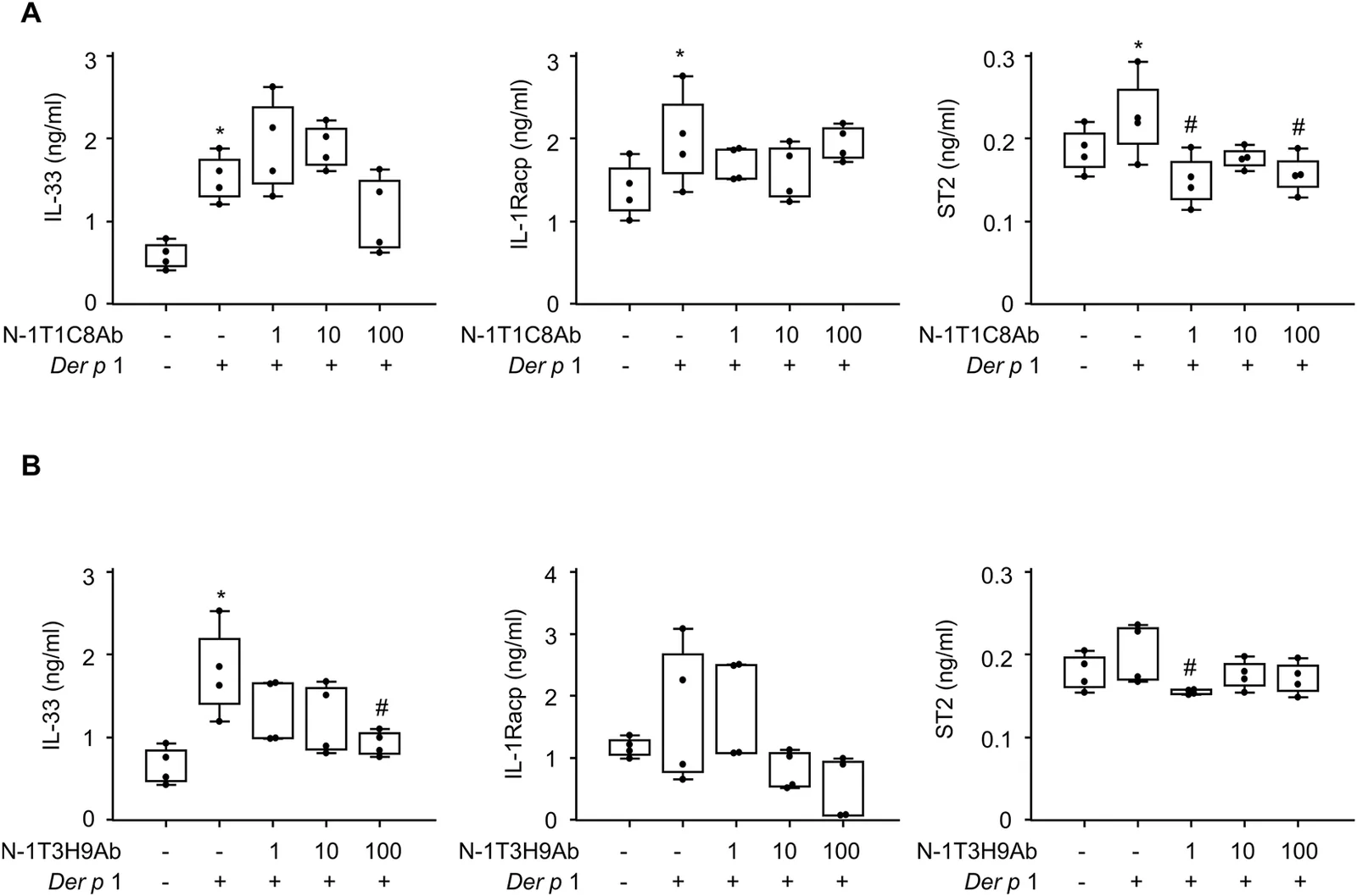
Anti-Nectin4 monoclonal antibody affects epithelial cell driven cytokine levels in NHBE cells. **(A)** Epithelial cell driven cytokine levels in NHBE cells after treatment with N-1T1C8Ab for 48 h following *Der p* 1 application for 8 h. **(B)** Epithelial cell driven cytokine levels in NHBE cells after treatment with N-1T3H9Ab for 48 h following *Der p* 1 application for 8 h. Data are means ± SEMs (n = 4). *p < 0.05 compared to control, ^#^p < 0.05 compared to *Der p* 1.

### Anti-Nectin4 monoclonal antibodies modulate MyD88/IL-1R-associated kinase (IRAK) signaling in NHBE cells treated with *Der p* 1

The expression levels of MyD88, IRAK1, IRAK4, and TRAF6 (Figures 3A,E), as well as p-NF-κB/NF-κB, p-JNK/JNK, p-p38/p38, and p-ERK1/2/ERK1/2 (Figures 3C,G), were assessed in NHBE cells after *Der p* 1 exposure. N-1T1C8Ab significantly reduced IRAK4 expression at 1, 10, and 100 μg/mL, whereas MyD88, IRAK1, TRAF6, NF-κB, JNK, p38, and ERK1/2 showed decreasing trends without statistical significance (Figures 3A–D). In contrast, N-1T3H9Ab significantly reduced MyD88, IRAK1, and TRAF6 expression at 100 μg/mL and IRAK4 expression at 10 μg/mL. NF-κB and JNK showed decreasing trends following N-1T3H9Ab treatment, whereas p38 and ERK1/2 showed no consistent reduction (Figures 3E–H).

**FIGURE 3:**
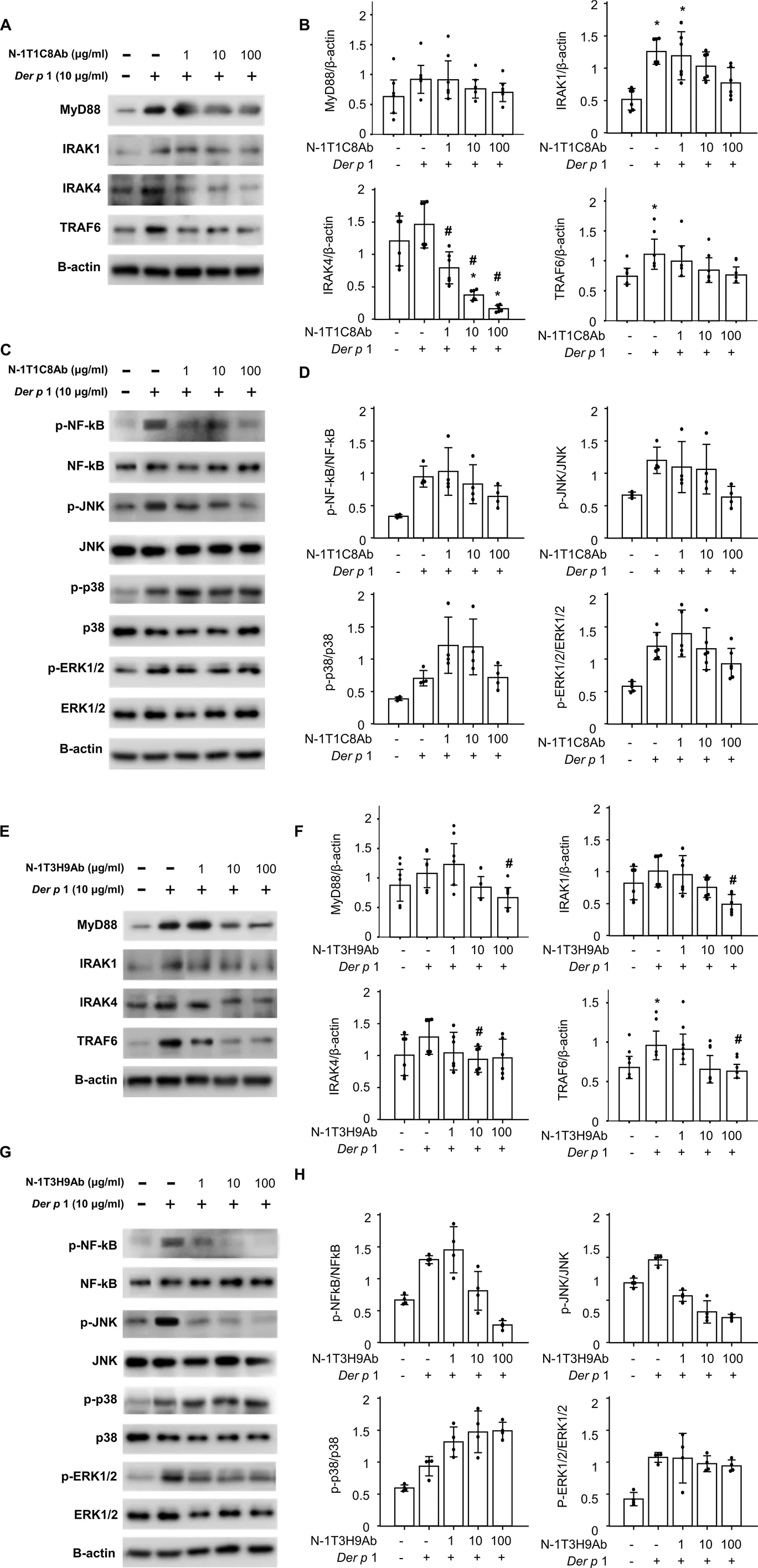
Anti-Nectin4 monoclonal antibody controls the expression of MyD88 signaling pathway in NHBE cells. **(A, C)** Expression of the MyD88 signaling pathway of NHBE cells after treatment with N-1T1C8Ab for 48 h following *Der p* 1 application for 8 h. **(B, D)** Densitometry of the bands obtained on three different individuals western blots. **(E, G)** Expression of the MyD88 signaling pathway of NHBE cells after treatment with N-1T3H9Ab for 48 h following *Der p* 1 application for 8 h. **(F, H)** Densitometry of the bands obtained on three different individuals western blots. The values were normalized to those of β-actin. Data are means ± SEMs. *p < 0.05 compared to control, ^#^p < 0.05 compared to *Der p* 1.

### Anti-Nectin4 monoclonal antibodies modulate Afadin/Rac1 signaling in NHBE cells treated with *Der p* 1

Nectin4/Afadin and Rac1 pathway protein levels were increased in NHBE cells after *Der p* 1 exposure (Figures 4A,C). However, both the expression of Nectin4/Afadin and the levels of the activated form of Rac1 (Rac1-GTP) were decreased after N-1T1C8Ab or N-1T3H9Ab treatment (Figures 4B,D), indicating that Nectin4 blockade was associated with altered Afadin/Rac1 signaling following *Der p* 1 exposure.

**FIGURE 4:**
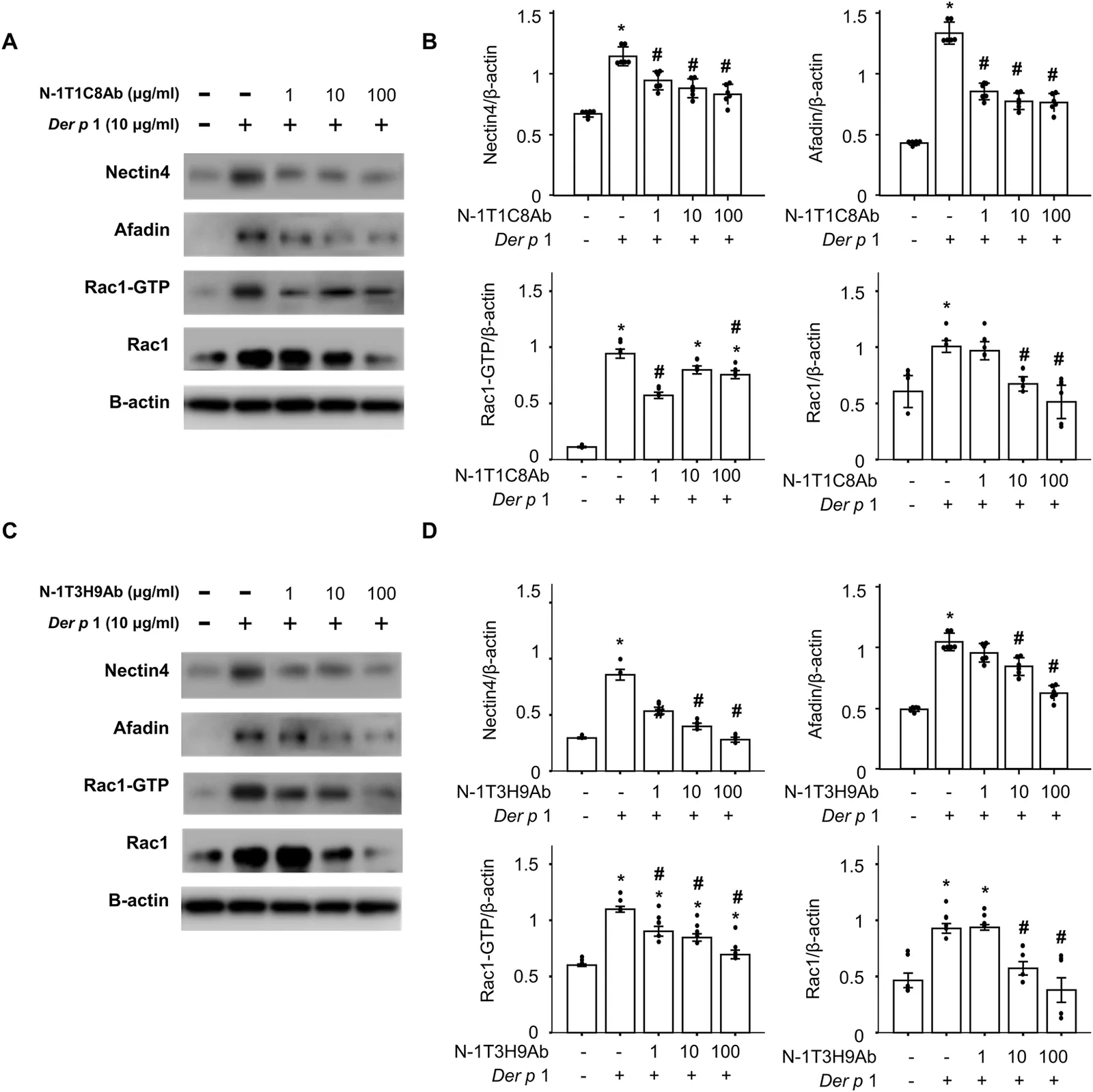
Anti-Nectin4 monoclonal antibody controls the expression of Afadin and Rac1 pathway in NHBE cells. **(A)** Expression of the Afadin and Rac1 pathway of NHBE cells after treatment with N-1T1C8Ab for 48 h following *Der p* 1 application for 8 h. **(B)** Densitometry of the bands obtained on three different individuals western blots. **(C)** Expression of the Afadin and Rac1 pathway of NHBE cells after treatment with N-1T3H9Ab for 48 h following *Der p* 1 application for 8 h. **(D)** Densitometry of the bands obtained on three different individuals western blots. The values were normalized to those of β-actin. Data are means ± SEMs. *p < 0.05 compared to control, ^#^p < 0.05 compared to *Der p* 1.

### Anti-Nectin4 monoclonal antibodies attenuate airway hyperresponsiveness and lung inflammation in asthmatic mice

In the OVA-induced asthma model, mice were treated with N-1T1C8Ab or N-1T3H9Ab as shown in Figure 5A. The OVA group exhibited significantly increased airway resistance (Rn) in response to inhaled methacholine concentrations (MCh; 0–50 mg/mL) compared to the control group, whereas treatment with N-1T1C8Ab or N-1T3H9Ab attenuated this response (Figure 5B). BALF analysis showed a significantly increased total cell numbers and counts of macrophages, eosinophils, neutrophils, and lymphocytes in the OVA group. Anti-Nectin4 antibody treatment reduced total inflammatory cell counts, including eosinophils and neutrophils (Figure 5C). Histopathological examination further revealed prominent peribronchial inflammatory cell infiltration in OVA-exposed mice, which was significantly reduced following N-1T1C8Ab or N-1T3H9Ab treatment (Figures 5D,E). OVA exposure also increased goblet cell hyperplasia and mucus production, collagen deposition, and mast cell accumulation, whereas these pathological changes were attenuated by anti-Nectin4 antibody treatment (Figures 5F–H). Collectively, these results show that anti-Nectin4 antibody treatment ameliorated multiple features of experimental asthma, including airway hyperresponsiveness, inflammatory cell infiltration, and airway remodeling.

**FIGURE 5:**
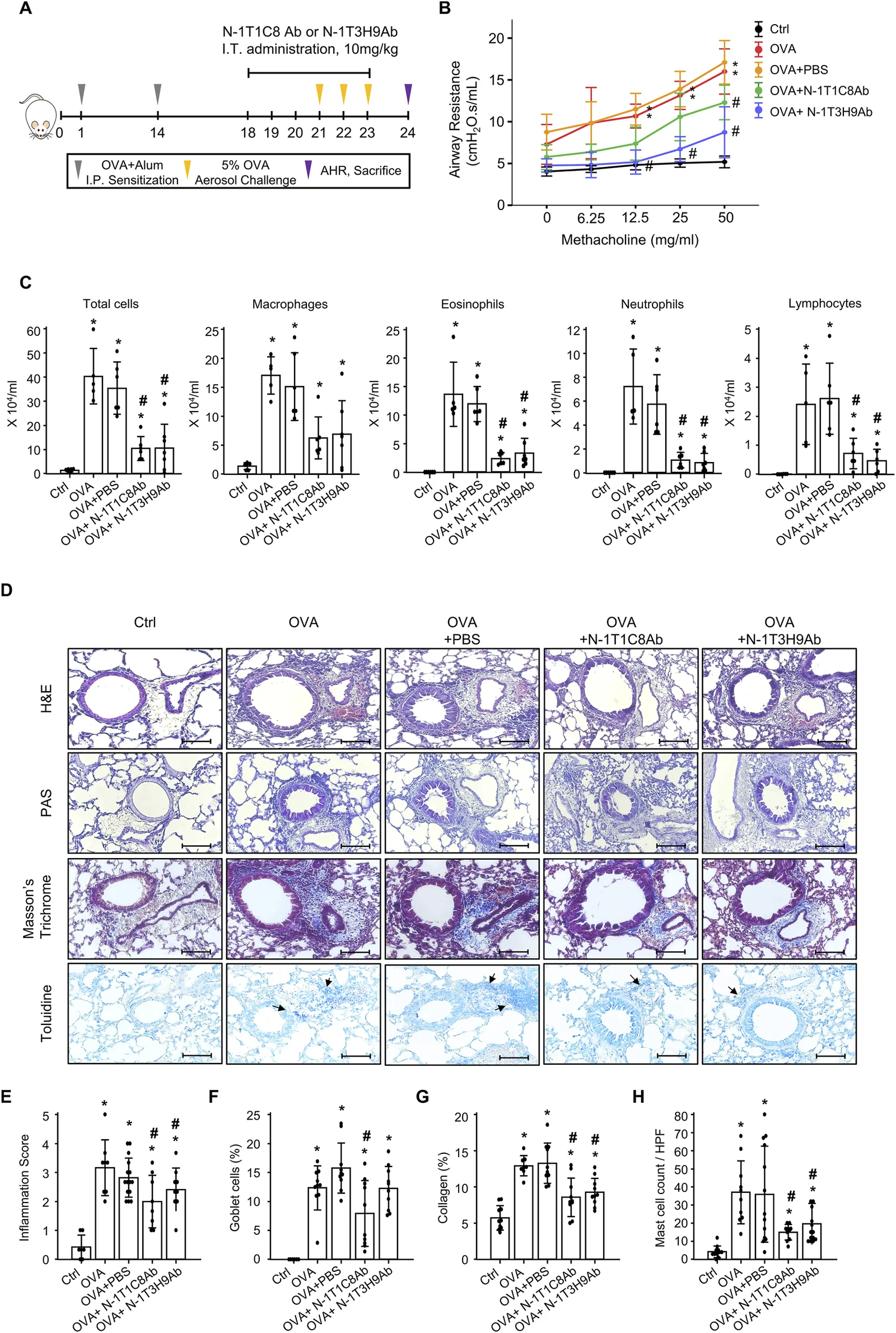
The OVA-induced asthma mouse model with Anti-Nectin4 monoclonal antibody treatment. **(A)** The establishment of mouse asthma model. **(B)** Airway resistance measured after inhaling increasing doses of methacholine. **(C)** Numbers of all cells, macrophages, eosinophils, and neutrophils per milliliter BALF. **(D)** Representative images of H&E, Masson’s trichrome, PAS, and Toluidin-stained, paraffin-embedded lung sections of mice (scale bar = 50 μm). **(E–H)** Bar graphs summarize the means ± SEMs of the histological scores of inflammation grade, fibrosis, and number of goblet cells and mast cells. Data are means ± SEMs (n = 8–10 per group). *p < 0.05 compared to control, ^#^p < 0.05 compared to OVA.

### Anti-Nectin4 monoclonal antibodies modulate inflammatory cytokines and IL-33-related signaling components in asthmatic mice

The levels of IL-4 and TNF-α were significantly increased in the OVA group compared with the control group, whereas IL-5 and IFN-γ showed no significant differences (Figure 6A). Treatment with N-1T1C8Ab or N-1T3H9Ab significantly attenuated the OVA-induced increase in IL-4, while no significant changes were observed in IL-5, TNF-α, or IFN-γ following antibody treatment (Figure 6A). In addition, IL-33 and its receptor components, ST2 and IL-1RAcP, were significantly elevated in the OVA group compared with the control group. Anti-Nectin4 antibody treatment reduced the levels of these IL-33-related signaling components (Figure 6B). Overall, anti-Nectin4 antibody treatment was associated with selective modulation of inflammatory cytokines and IL-33-related signaling components in the OVA-induced asthma model.

**FIGURE 6:**
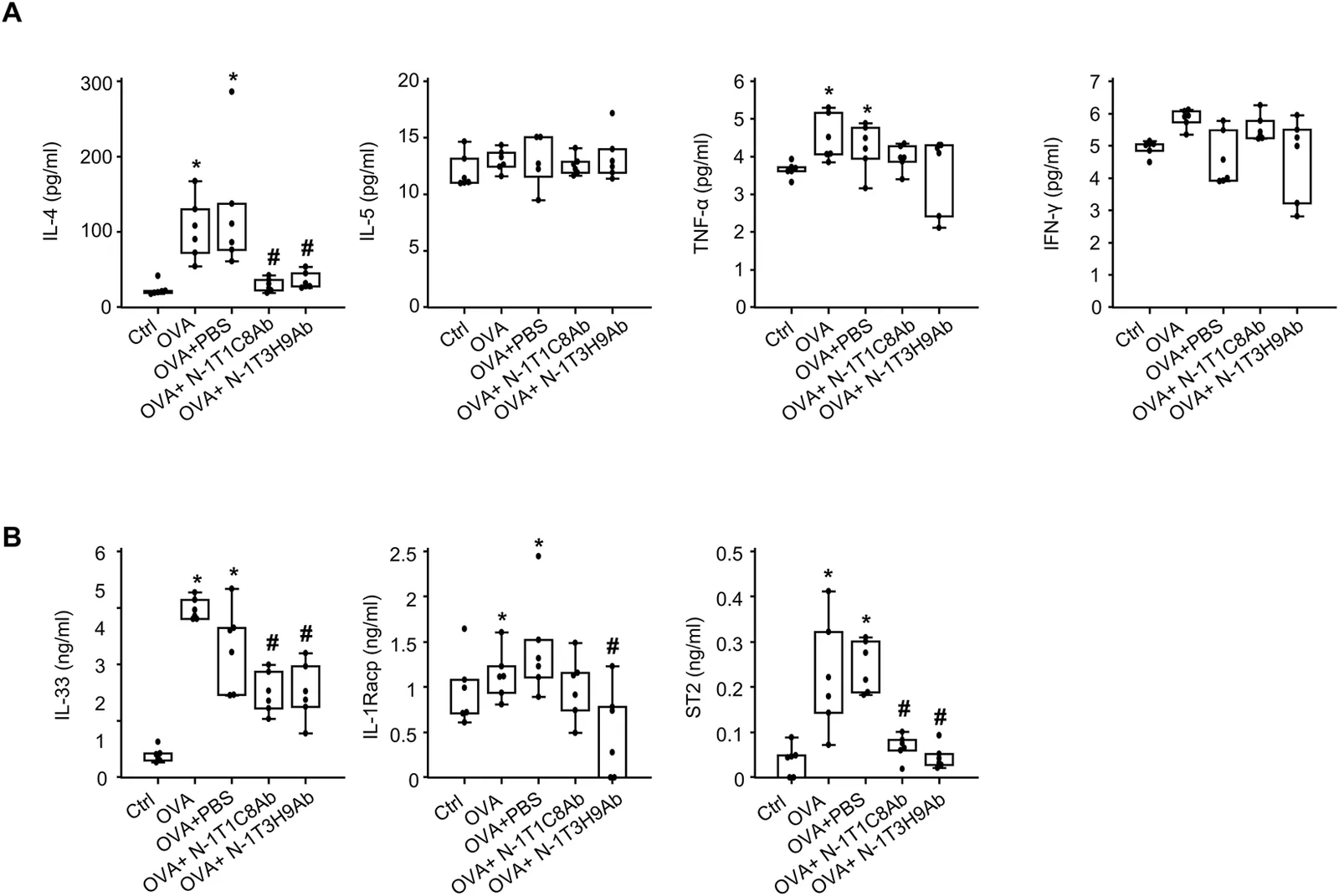
Anti-Nectin4 monoclonal antibody affects inflammatory cytokines in OVA-induced asthma mouse model. **(A)** Th1/Th2 cytokine levels. **(B)** epithelial cell driven cytokine levels in BALF based on ELISA. Data are means ± SEMs (n = 8–10 per group). *p < 0.05 compared to control, ^#^p < 0.05 compared to OVA.

### Anti-Nectin4 monoclonal antibodies modulate MyD88/IRAK-related signaling in asthmatic mice

To determine whether the signaling changes observed in NHBE cells were also present *in vivo*, we assessed MyD88/IRAK- and MAPK-related signaling proteins in lung tissues. MyD88, IRAK4, and TRAF6 were significantly increased in the OVA group compared with the control group, whereas IRAK1 showed an increasing trend (Figures 7A,B). Treatment with either N-1T1C8Ab or N-1T3H9Ab significantly reduced MyD88, IRAK1, IRAK4, and TRAF6 compared with the OVA group. In addition, the phosphorylation levels of NF-κB, p38, and ERK1/2 were significantly increased in the OVA group, whereas JNK phosphorylation showed an increasing trend (Figures 7C,D). Among these signaling proteins, ERK1/2 phosphorylation was significantly reduced by both anti-Nectin4 antibodies, whereas NF-κB, JNK, and p38 phosphorylation was not significantly altered following antibody treatment.

**FIGURE 7:**
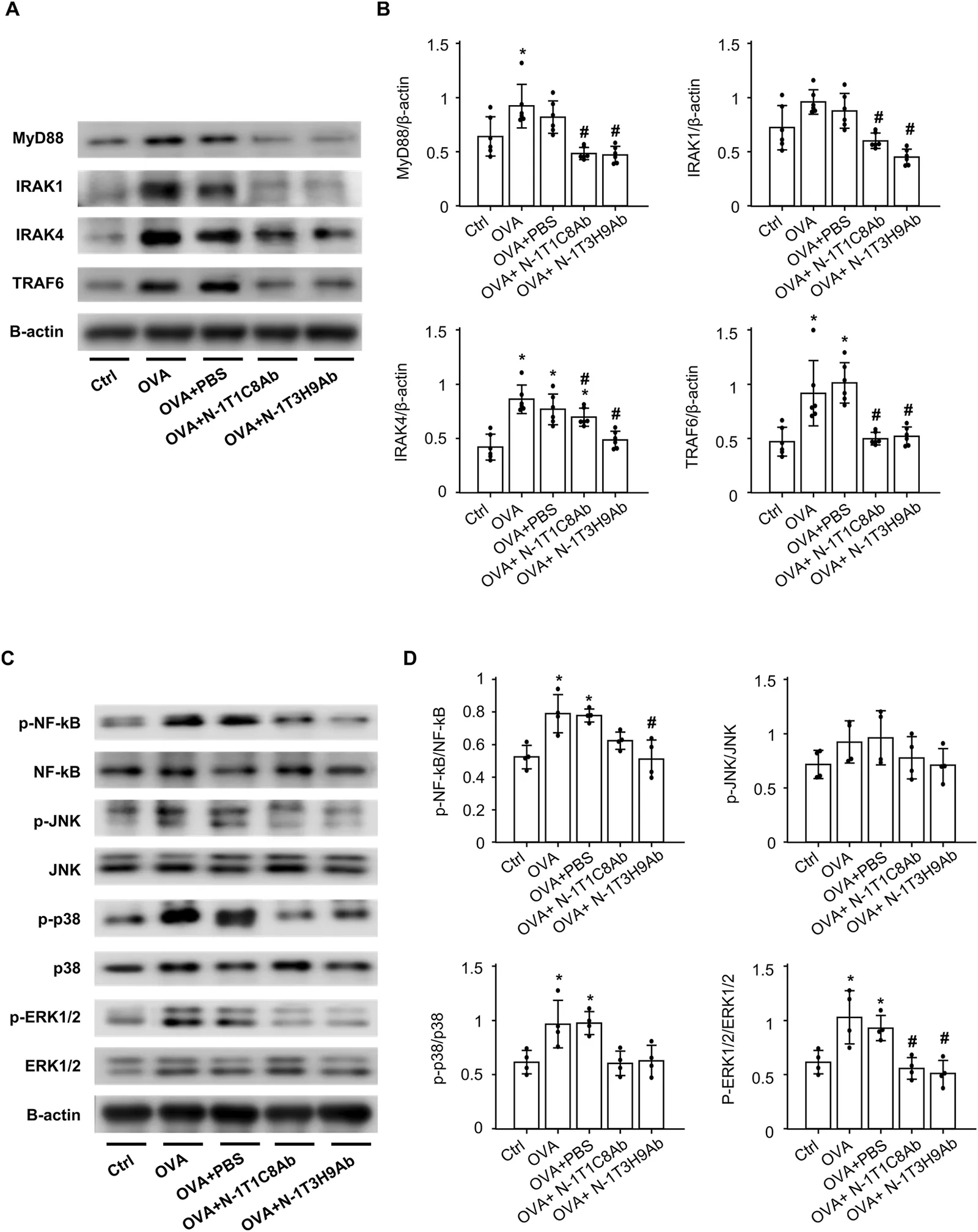
Anti-Nectin4 monoclonal antibody controls the expression of MyD88 signaling pathway in OVA-induced asthma mouse model. **(A, C)** Expression of the MyD88 signaling pathway. **(B, D)** Densitometry of the bands obtained on three different individuals western blots. The values were normalized to those of β-actin. Data are means ± SEMs (n = 6 per group). *p < 0.05 compared to control, ^#^p < 0.05 compared to OVA.

### Anti-Nectin4 monoclonal antibodies modulate Afadin/Rac1 signaling in asthmatic mice

Nectin4 and Afadin expression and Rac1-GTP levels were increased in the lungs of OVA-exposed mice compared with the control group (Figures 8A,B). Treatment with N-1T1C8Ab or N-1T3H9Ab reduced Nectin4 and Afadin expression as well as Rac1-GTP levels. Consistent with the Western blot results, double immunofluorescence staining showed increased Nectin4 and Rac1-GTP signals in the lungs of OVA-exposed mice, which were reduced following treatment with either anti-Nectin4 antibody (Figures 8C,D).

**FIGURE 8:**
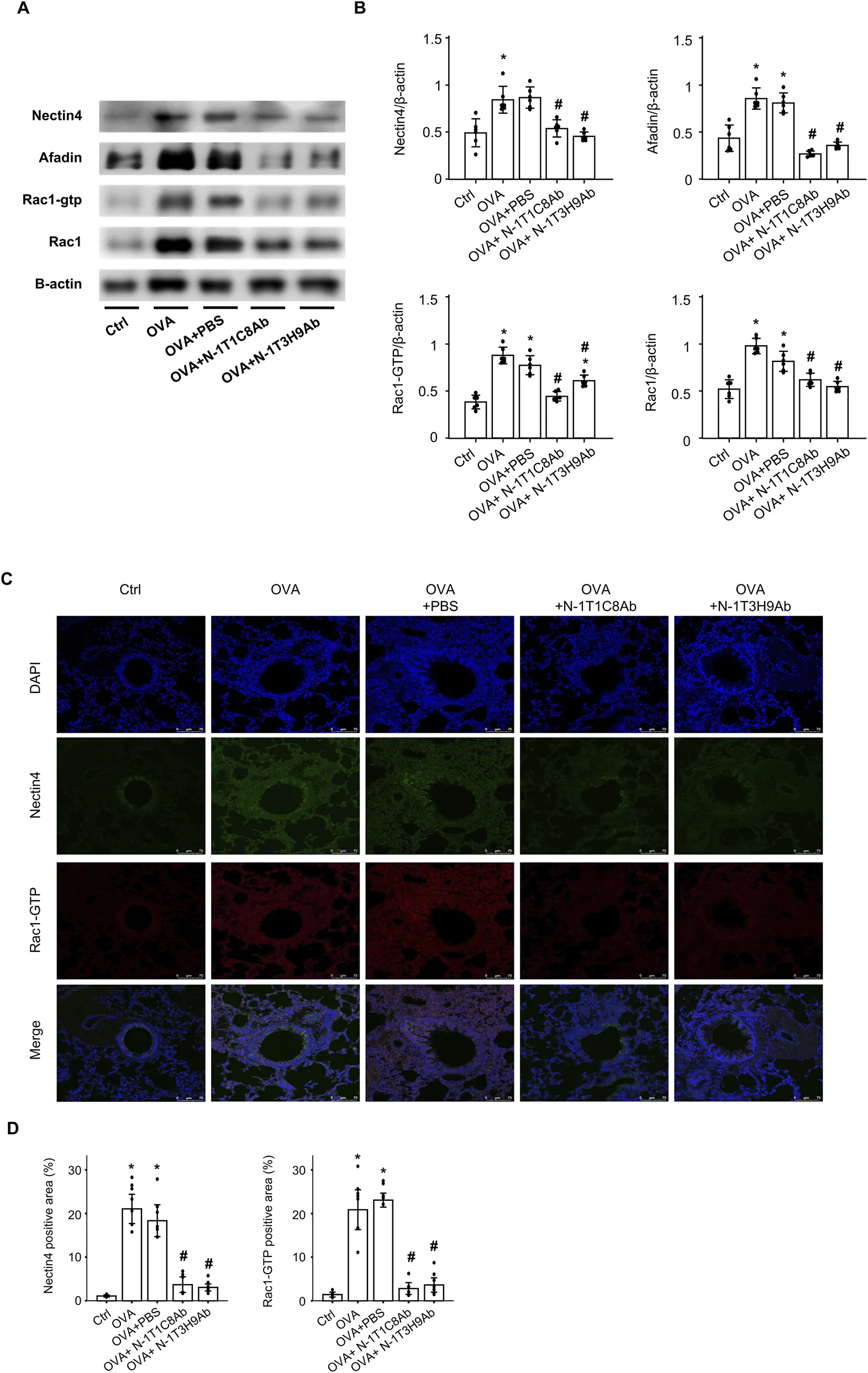
Anti-Nectin4 monoclonal antibody controls the expression of Afadin and Rac1 pathway in OVA-induced asthma mouse model. **(A)** Expression of the Nectin4/Afadin and the Rac1 pathway. **(B)** Densitometry of the bands obtained on three different individuals western blots. The values were normalized to those of β-actin. **(C)** Double immunofluorescence images of Nectin4 and Rac1-GTP in lung of the mice (scale bar = 100 μm). **(D)** Quantitative analysis of Nectin4 and Rac1-GTP expression level. Data are means ± SEMs (n = 6 per group). *p < 0.05 compared to control, ^#^p < 0.05 compared to OVA.

## Discussion

The study was designed to determine whether anti–Nectin4 antibody reduces airway inflammation and hyperreactivity in a murine model of allergen-induced asthma. We observed that intratracheal administration of anti-Nectin4 antibody before allergen challenge reduced IL-4 levels, airway resistance, and inflammatory cell infiltration in asthmatic mice. These effects were accompanied by changes in Nectin4/Afadin–Rac1 signaling and selected components of IL-33- and MyD88/IRAK-related signaling in bronchial epithelial cells and asthmatic mice. However, the present data demonstrate associations and do not establish that modulation of these pathways is required for the observed effects of Nectin4 blockade.

Junctional proteins are essential components of cell-cell adhesion in epithelial tissues, playing a pivotal role in maintaining the integrity of the epithelial barrier (Nakao, 2020). In asthma, disruption of epithelial junctional proteins increases airway permeability and facilitates the penetration of allergens and inflammatory mediators into the airway tissue (Georas and Rezaee, 2014). This process promotes inflammatory cell recruitment and contributes to airway hyperresponsiveness and chronic inflammation. Several junctional proteins, including claudins, occludin, and cadherins, have been reported to regulate epithelial barrier function and inflammatory responses (Lee et al., 2018; Moon et al., 2015; Lee et al., 2021; Tan et al., 2019; Xiao et al., 2011). Consistent with the importance of epithelial barrier integrity in asthma, *Der p* 1 exposure reduced TEER in NHBE cells in the present study, whereas anti-Nectin4 antibody treatment attenuated this reduction. This finding suggests that Nectin4 blockade may be associated with preservation of epithelial barrier function, although individual tight junction proteins were not evaluated.

Nectin4 is an immunoglobulin-like cell adhesion molecule that functions at both adherens and tight junctions (Takai et al., 2008b). It contributes to cell–cell adhesion and plays an important role in maintaining epithelial tissue integrity and regulating intercellular signaling pathways (Samanta and Almo, 2015). Through interactions with adaptor proteins such as Afadin, Nectin4 may influence intracellular signaling pathways involved in cellular proliferation, inflammation, and cytoskeletal organization. In the present study, anti-Nectin4 antibody treatment was accompanied by changes in Nectin4/Afadin expression and Rac1 activation in both NHBE cells and asthmatic mice, supporting a potential association between Nectin4-related junctional signaling and the observed effects of antibody treatment.

Our previous study identified Nectin4 as a potential biomarker for asthma. Nectin4 deletion reduced airway inflammation and inflammatory cytokine production and was associated with reduced activation of the Src/Rac1 signaling pathway (Lee et al., 2022). Together with the present findings, these observations further support a role for Nectin4 in airway inflammatory responses, although the specific contribution of Afadin/Rac1 signaling to the effects of anti-Nectin4 antibodies remains to be determined.

To date, Nectin4 is highly expressed in a variety of tumors while showing minimal expression in normal tissues, making it an attractive target for antibody-based cancer therapies, including antibody-drug conjugates (ADCs) (Challita-Eid et al., 2016; Khosravanian et al., 2024; Halford et al., 2021). Notably, the anti-Nectin4 antibody–drug conjugate Enfortumab vedotin has demonstrated significant clinical efficacy in patients with advanced urothelial carcinoma, indicating that Nectin4 is a therapeutically actionable target in human disease (Powles et al., 2021; Challita-Eid et al., 2016). Although Nectin4–targeted therapy has not yet been explored in asthma, these findings provide a rationale for investigating Nectin4 blockade as a strategy to modulate airway inflammation. Unlike Nectin4-targeted ADCs used in oncology, the antibodies investigated here were evaluated for their potential to modulate Nectin4-associated epithelial and inflammatory responses rather than to deliver a cytotoxic payload. In the present study, anti-Nectin4 monoclonal antibody treatment was associated with improved epithelial barrier function and attenuation of airway inflammation *in vitro* and *in vivo*. Changes in Nectin4/Afadin–Rac1 signaling and selected components of IL-33/MyD88-related signaling were observed following antibody treatment. Administration of N-1T1C8Ab or N-1T3H9Ab reduced airway inflammation, airway resistance, and the number of immune cells in bronchoalveolar lavage fluid. Histological analysis further demonstrated decreased inflammatory cell infiltration, goblet cell hyperplasia, fibrosis, and mast cell accumulation in lung tissues. These findings suggest that anti-Nectin4 treatment may attenuate airway inflammation and associated pathological features in this preclinical asthma model.

Currently, several monoclonal antibodies are available for asthma treatment, including anti-IgE, anti-IL-5, anti-IL-5 receptor α, and anti-IL-4 receptor α antibodies (Abe et al., 2021). Although these biologics improve disease control in many patients, their clinical efficacy varies depending on asthma phenotype and inflammatory profile. Consequently, additional therapeutic targets, particularly epithelial-derived alarmins such as thymic stromal lymphopoietin (TSLP), IL-25, and IL-33, are actively being investigated (Abe et al., 2021). Because IL-33 participates in both type 2 and non-type 2 inflammatory pathways, targeting IL-33-related signaling may provide broader therapeutic benefits than strategies focusing exclusively on type 2 inflammation (Calderon et al., 2023). In contrast to these cytokine- or cytokine receptor-targeted approaches, Nectin4 is an epithelial cell-adhesion molecule, and its targeting may represent a distinct strategy involving epithelial barrier function and associated inflammatory responses. However, further studies are required to determine whether Nectin4 targeting provides therapeutic advantages over currently available or emerging biologics.

IL-33 plays an important role in the initiation of allergic airway inflammation by activating immune cells such as eosinophils, mast cells, and innate lymphoid cells (Calderon et al., 2023; Stolarski et al., 2010; Chen et al., 2017). Binding of IL-33 to the ST2 receptor recruits the adaptor protein MyD88, leading to activation of downstream inflammatory signaling pathways including NF-κB and MAPKs (Agoro et al., 2016; McGettrick and O’Neill, 2004). In the present study, anti-Nectin4 antibody treatment was associated with changes in IL-33-related signaling components and MyD88-related signaling. However, the effects were not consistent across all signaling components or antibody concentrations. Therefore, these findings support an association between Nectin4 blockade and IL-33/MyD88-related signaling rather than establishing suppression of this pathway as a primary mechanism of action.

This study has several limitations. Detailed structural changes in the airway epithelium and the expression of tight junction proteins such as claudins, ZO-1, and occludin were not evaluated. In addition, although anti-Nectin4 treatment was associated with changes in IL-33/MyD88-related signaling, the present study does not establish whether Nectin4 directly regulates IL-33 or whether these changes occur secondarily to improved epithelial barrier function and reduced airway inflammation. Furthermore, the hierarchical relationship between Nectin4/Afadin–Rac1 signaling and IL-33/MyD88-related signaling remains unclear. Thus, whether these pathways are independently modulated or functionally interconnected following Nectin4 blockade cannot be determined from the current data. Further mechanistic studies are needed to clarify these relationships and determine their contribution to the observed effects of anti-Nectin4 antibodies.

## Conclusion

In conclusion, our findings show that anti-Nectin4 antibody treatment is associated with improved epithelial barrier function and reduced eosinophilic airway inflammation and airway hyperresponsiveness. Further preclinical studies are warranted to characterize the mechanisms, safety, and pharmacological properties of anti-Nectin4 antibodies before their potential clinical application in asthma can be considered.

## Data Availability

The original contributions presented in the study are included in the article/supplementary material, further inquiries can be directed to the corresponding author.

## References

[B1] Abe Y. Suga Y. Fukushima K. Ohata H. Niitsu T. Nabeshima H. (2021). Advances and challenges of antibody therapeutics for severe bronchial asthma. Int. J. Mol. Sci. 23, 83. 10.3390/ijms23010083 35008504 PMC8744863

[B2] Agoro R. Piotet-Morin J. Palomo J. Michaudel C. Vigne S. Maillet I. (2016). IL-1R1-MyD88 axis elicits papain-induced lung inflammation. Eur. J. Immunol. 46, 2531–2541. 10.1002/eji.201646366 27569535

[B3] Bleecker E. R. Menzies-Gow A. N. Price D. B. Bourdin A. Sweet S. Martin A. L. (2020). Systematic literature review of systemic corticosteroid use for asthma management. Am. J. Respir. Crit. Care Med. 201, 276–293. 10.1164/rccm.201904-0903SO 31525297 PMC6999108

[B4] Calderon A. A. Dimond C. Choy D. F. Pappu R. Grimbaldeston M. A. Mohan D. (2023). Targeting interleukin-33 and thymic stromal lymphopoietin pathways for novel pulmonary therapeutics in asthma and COPD. Eur. Respir. Rev. 32, 220144. 10.1183/16000617.0144-2022 36697211 PMC9879340

[B5] Cayrol C. Girard J. P. (2018). Interleukin-33 (IL-33): a nuclear cytokine from the IL-1 family. Immunol. Rev. 281, 154–168. 10.1111/imr.12619 29247993

[B6] Challita-Eid P. M. Satpayev D. Yang P. An Z. Morrison K. Shostak Y. (2016). Enfortumab vedotin antibody-drug conjugate targeting Nectin-4 is a highly potent therapeutic agent in multiple preclinical cancer models. Cancer. Res. 76, 3003–3013. 10.1158/0008-5472.CAN-15-1313 27013195

[B7] Chen C. C. Kobayashi T. Iijima K. Hsu F. C. Kita H. (2017). IL-33 dysregulates regulatory T cells and impairs established immunologic tolerance in the lungs. J. Allergy Clin. Immunol. 140, 1351–1363.e7. 10.1016/j.jaci.2017.01.015 28196763 PMC5554091

[B8] Cho K. A. Suh J. W. Sohn J. H. Park J. W. Lee H. Kang J. L. (2012). IL-33 induces Th17-mediated airway inflammation via mast cells in ovalbumin-challenged mice. Am. J. Physiol. Lung. Cell. Mol. Physiol. 302, L429–L440. 10.1152/ajplung.00252.2011 22180658

[B9] Deng H. Shi H. Chen L. Zhou Y. Jiang J. (2019). Over-expression of Nectin-4 promotes progression of esophageal cancer and correlates with poor prognosis of the patients. Cancer. Cell. Int. 19, 106. 10.1186/s12935-019-0824-z 31043861 PMC6480822

[B10] Fabre-Lafay S. Monville F. Garrido-Urbani S. Berruyer-Pouyet C. Ginestier C. Reymond N. (2007). Nectin-4 is a new histological and serological tumor associated marker for breast cancer. BMC. Cancer. 7, 73. 10.1186/1471-2407-7-73 17474988 PMC1868744

[B11] Garg D. Que L. G. Ingram J. L. (2024). Effects of biological therapies on patients with Type-2 high asthma and comorbid obesity. Front. Pharmacol. 14, 1315540. 10.3389/fphar.2023.1315540 38259298 PMC10800376

[B12] Georas S. N. Rezaee F. (2014). Epithelial barrier function: at the front line of asthma immunology and allergic airway inflammation. J. Allergy Clin. Immunol. 134, 509–520. 10.1016/j.jaci.2014.05.049 25085341 PMC4170838

[B13] Global Initiative for Asthma (GINA) (2023). Difficult-to-treat and severe asthma in adolescent and adult patients. Available online at: https://ginasthma.org/wp-content/uploads/2023/09/GINA-Severe-Asthma-Guide-2023-WEB-WMS.pdf (Accessed August 28, 2026).

[B14] Global Initiative For Chronic Obstructive Lung Disease (2023). World asthma day 2023. Available online at: https://goldcopd.org/world-asthma-day-2023/ (Accessed September 27, 2023).

[B15] Günther S. Deredge D. Bowers A. L. Luchini A. Bonsor D. A. Beadenkopf R. (2017). IL-1 family cytokines use distinct molecular mechanisms to signal through their shared Co-receptor. Immunity 47, 510–523.e4. 10.1016/j.immuni.2017.08.004 28930661 PMC5849085

[B16] Halford Z. Anderson M. K. Clark M. D. (2021). Enfortumab vedotin-ejfv: a first-in-class Anti-Nectin-4 antibody-drug conjugate for the management of urothelial carcinoma. Ann. Pharmacother. 55, 772–782. 10.1177/1060028020960402 32945172

[B17] Inselman J. W. Jeffery M. M. Maddux J. T. Shah N. D. Rank M. A. (2020). Trends and disparities in asthma biologic use in the United States. J. Allergy Clin. Immunol. Pract. 8, 549–554.e1. 10.1016/j.jaip.2019.08.024 31472294 PMC7012665

[B18] Ishii Y. Itoh K. Morishima Y. Kimura T. Kiwamoto T. Iizuka T. (2005). Transcription factor Nrf2 plays a pivotal role in protection against elastase-induced pulmonary inflammation and emphysema. J. Immunol. 175, 6968–6975. 10.4049/jimmunol.175.10.6968 16272357

[B19] Khosravanian M. J. Mirzaei Y. Mer A. H. Keyhani-Khankahdani M. Abdinia F. S. Misamogooe F. (2024). Nectin-4-directed antibody-drug conjugates (ADCs): spotlight on preclinical and clinical evidence. Life. Sci. 352, 122910. 10.1016/j.lfs.2024.122910 39002610

[B20] Kim H. Ellis A. K. Fischer D. Noseworthy M. Olivenstein R. Chapman K. R. (2017). Asthma biomarkers in the age of biologics. Allergy Asthma.Clin. Immunol. 13, 48. 10.1186/s13223-017-0219-4 29176991 PMC5691861

[B21] Lee P. H. Kim B. G. Lee S. H. Lee J. H. Park S. W. Kim D. J. (2018). Alteration in claudin-4 contributes to airway inflammation and responsiveness in asthma. Allergy Asthma. Immunol. Res. 10, 25–33. 10.4168/aair.2018.10.1.25 29178675 PMC5705480

[B22] Lee Y. G. Lee S. H. Hong J. Lee P. H. Jang A. S. (2021). Titanium dioxide particles modulate epithelial barrier protein, claudin 7 in asthma. Mol. Immunol. 132, 209–216. 10.1016/j.molimm.2021.01.004 33483086

[B23] Lee P. H. Choi S. M. An M. H. Hwang D. Y. Park S. Baek A. R. (2022). Nectin4 is a potential therapeutic target for asthma. Front. Immunol. 13, 1049900. 10.3389/fimmu.2022.1049900 36457999 PMC9707334

[B24] Liew F. Y. Girard J. P. Turnquist H. R. (2016). Interleukin-33 in health and disease. Nat. Rev. Immunol. 16, 676–689. 10.1038/nri.2016.95 27640624

[B25] Liu X. Hammel M. He Y. Tainer J. A. Jeng U. S. Zhang L. (2013). Structural insights into the interaction of IL-33 with its receptors. Proc. Natl. Acad. Sci. U. S. A. 110, 14918–14923. 10.1073/pnas.1308651110 23980170 PMC3773798

[B26] Louis R. Satia I. Ojanguren I. Schleich F. Bonini M. Tonia T. (2022). European respiratory society guidelines for the diagnosis of asthma in adults. Eur. Respir. J. 60, 2101585. 10.1183/13993003.01585-2021 35169025

[B27] Luskin A. T. Antonova E. N. Broder M. S. Chang E. Y. Omachi T. A. Ledford D. K. (2016). Health care resource use and costs associated with possible side effects of high oral corticosteroid use in asthma: a claims-based analysis. Clinicoecon. Outcomes. Res. 8, 641–648. 10.2147/CEOR.S115025 27822075 PMC5087584

[B28] McGettrick A. F. O’Neill L. A. (2004). The expanding family of MyD88-like adaptors in toll-like receptor signal transduction. Mol. Immunol. 41, 577–582. 10.1016/j.molimm.2004.04.006 15219996

[B29] Menzies-Gow A. Corren J. Bourdin A. Chupp G. Israel E. Wechsler M. E. (2021). Tezepelumab in adults and adolescents with severe, uncontrolled asthma. N. Engl. J. Med. 384, 1800–1809. 10.1056/NEJMoa2034975 33979488

[B30] Molofsky A. B. Savage A. K. Locksley R. M. (2015). Interleukin-33 in tissue homeostasis, injury, and inflammation. Immunity 42, 1005–1019. 10.1016/j.immuni.2015.06.006 26084021 PMC4471869

[B31] Moon K. Y. Lee P. H. Kim B. G. Park C. S. Leikauf G. D. Jang A. S. (2015). Claudin 5 in a murine model of allergic asthma: its implication and response to steroid treatment. J. Allergy Clin. Immunol. 136, 1694–1696.e5. 10.1016/j.jaci.2015.08.004 26409663

[B32] Nakao A. (2020). Circadian regulation of the biology of allergic disease: Clock disruption can promote allergy. Front. Immunol. 11, 1237. 10.3389/fimmu.2020.01237 32595651 PMC7304491

[B33] Nishiwada S. Sho M. Yasuda S. Shimada K. Yamato I. Akahori T. (2015). Nectin-4 expression contributes to tumor proliferation, angiogenesis and patient prognosis in human pancreatic cancer. J. Exp. Clin. Cancer. Res. 34, 30. 10.1186/s13046-015-0144-7 25888293 PMC4387735

[B34] Pichery M. Mirey E. Mercier P. Lefrancais E. Dujardin A. Ortega N. (2012). Endogenous IL-33 is highly expressed in mouse epithelial barrier tissues, lymphoid organs, brain, embryos, and inflamed tissues: *in situ* analysis using a novel Il-33-LacZ gene trap reporter strain. J. Immunol. 188, 3488–3495. 10.4049/jimmunol.1101977 22371395

[B35] Powles T. Rosenberg J. E. Sonpavde G. P. Loriot Y. Durán I. Lee J. L. (2021). Enfortumab vedotin in previously treated advanced urothelial carcinoma. N. Engl. J. Med. 384, 1125–1135. 10.1056/NEJMoa2035807 33577729 PMC8450892

[B36] Ross M. M. Chung Y. Carr T. Ambrose C. S. Lindsley A. W. Collacott H. (2024). Patient and clinician preferences for biologic treatments for severe uncontrolled asthma: a discrete choice experiment (DCE). J. Asthma. 1, 1–13. 10.1080/02770903.2024.2380520 39007899

[B37] Saluja R. Hawro T. Eberle J. Church M. K. Maurer M. (2014). Interleukin-33 promotes the proliferation of mouse mast cells through ST2/MyD88 and p38 MAPK-dependent and Kit-independent pathways. J. Biol. Regul. Homeost. Agents. 28, 575–585. 25620169

[B38] Saluja R. Ketelaar M. E. Hawro T. Church M. K. Maurer M. Nawijn M. C. (2015). The role of the IL-33/IL-1RL1 axis in mast cell and basophil activation in allergic disorders. Mol. Immunol. 63, 80–85. 10.1016/j.molimm.2014.06.018 25017307

[B39] Samanta D. Almo S. C. (2015). Nectin family of cell-adhesion molecules: structural and molecular aspects of function and specificity. Cell. Mol. Life. Sci. 72, 645–658. 10.1007/s00018-014-1763-4 25326769 PMC11113404

[B40] Schmitz J. Owyang A. Oldham E. Song Y. Murphy E. McClanahan T. K. (2005). IL-33, an interleukin-1-like cytokine that signals via the IL-1 receptor-related protein ST2 and induces T helper type 2-associated cytokines. Immunity 23, 479–490. 10.1016/j.immuni.2005.09.015 16286016

[B41] Schneider E. Petit-Bertron A. F. Bricard R. Levasseur M. Ramadan A. Girard J. P. (2009). IL-33 activates unprimed murine basophils directly *in vitro* and induces their *in vivo* expansion indirectly by promoting hematopoietic growth factor production. J. Immunol. 183, 3591–3597. 10.4049/jimmunol.0900328 19684081

[B42] Siddharth S. Goutam K. Das S. Nayak A. Nayak D. Sethy C. (2017). Nectin-4 is a breast cancer stem cell marker that induces WNT/β-catenin signaling via Pi3k/Akt axis. Int. J. Biochem. Cell. Biol. 89, 85–94. 10.1016/j.biocel.2017.06.007 28600142

[B43] Stolarski B. Kurowska-Stolarska M. Kewin P. Xu D. Liew F. Y. (2010). IL-33 exacerbates eosinophil-mediated airway inflammation. J. Immunol. 185, 3472–3480. 10.4049/jimmunol.1000730 20693421

[B44] Takai Y. Miyoshi J. Ikeda W. Ogita H. (2008a). Nectins and nectin-like molecules: roles in contact inhibition of cell movement and proliferation. Nat. Rev. Mol. Cell. Biol. 9, 603–615. 10.1038/nrm2457 18648374

[B45] Takai Y. Ikeda W. Ogita H. Rikitake Y. (2008b). The immunoglobulin-like cell adhesion molecule nectin and its associated protein afadin. Annu. Rev. Cell. Dev. Biol. 24, 309–342. 10.1146/annurev.cellbio.24.110707.175339 18593353

[B46] Takano A. Ishikawa N. Nishino R. Masuda K. Yasui W. Inai K. (2009). Identification of nectin-4 oncoprotein as a diagnostic and therapeutic target for lung cancer. Cancer. Res. 69, 6694–6703. 10.1158/0008-5472.CAN-09-0016 19679554

[B47] Tan H. T. Hagner S. Ruchti F. Radzikowska U. Tan G. Altunbulakli C. (2019). Tight junction, mucin, and inflammasome-related molecules are differentially expressed in eosinophilic, mixed, and neutrophilic experimental asthma in mice. Allergy 74, 294–307. 10.1111/all.13619 30267575

[B48] Xiao C. Puddicombe S. M. Field S. Haywood J. Broughton-Head V. Puxeddu I. (2011). Defective epithelial barrier function in asthma. J. Allergy Clin. Immunol. 128, 549–556.e1. 10.1016/j.jaci.2011.05.038 21752437

[B49] Zhang Y. Liu S. Wang L. Wu Y. Hao J. Wang Z. (2016). A novel PI3K/AKT signaling axis mediates Nectin4-induced gallbladder cancer cell proliferation, metastasis and tumor growth. Cancer. Lett. 375, 179–189. 10.1016/j.canlet.2016.02.049 26949052

